# Excerpta

**Published:** 1880-08

**Authors:** 


### IODINE A SUBSTITUTE FOR QUINIA.*

BY FORDYCE GRINNELL M. D., MARYVILLE, TENNESSEE.

While occupying the position of Government physician at the Wichita Indian Agency, Indian Territory, I saw a statement quoted from the St. Petersburger Medical Dochenschrift on the value of Iodine as a substitute for quinia. The statement made by the Author, Dr. J. Nonodnitschauski, that "when given boldly, ten to twelve drops of the tinct. in a glass of sweetened water every eight hours, *Iodine will never rank second to quinia in the treatment of intermittent fevers*," the more forcibly, impressed me, because at the time malarious diseases were prevailing extensively, and while I had been using the sulphate of quinia at the rate of one ounce per day, my stock suddenly became exhausted, and no article ordinarily used as a substitute remained in the dispensary. Our distance from medical supplies rendered our condition, under these circumstances very embarrassing. Hence, the readiness with which we seized upon any suggestions which seemed to afford means to fight our great enemy—malaria.

Having then a good opportunity to test the value of the remedy, I

*Reprint from proof-sheets of Transactions Tennessee Medical Society.

began by following the plan suggested by Dr. Nonodnitschauski, that is, giving ten drops of the tinct. in one third glass of sweetened water, thrice daily to adults, children receiving proportional doses. The results far surpassed my most sanguine expectations.

Indeed, I thought the statement rather extravagant, that Iodine when given as above indicated, "will never rank second to quinia in the treatment of intermittent fevers."

Subsequent experience however, both in that country and in this, has led me to conclude that the anti-periodic powers of Iodine are superior to any other remedy of the materia medica save quinia, and that it is by far the best known substitute for quinia.

At that time I treated 135 cases of intermittent fever, 74 being males, and 61 females; these included children and in some instances infants. The quotidian and tertian types of the fever were the forms principally presented. I also treated four cases of diarrhœa and eight cases of neuralgia, each of malarial origin, using the same remedy, only adding astringents or opiates as indicated. One hundred and forty-seven cases were thus treated with the iodine, and the results were *fully equal to those treated with the sulphate of quinia.*

The remedy seemed to act almost as by magic, in many instances the paroxysms were not repeated after the medicine was given, though the doses were repeated for a day or two after the cessation of the fever. In cases of enlarged spleen there was a more speedy reduction in the size of that organ than when the sulphate of quinia was used.

One important item in its favor was the fact that it was much more agreeable to take than quinine, and this with a large part of our population proved a potent argument in its favor. The iodine at once became a more popular remedy than quinine, with the masses of our people.

The nationality of those treated embraced the white, the Indian and the negro races.

Knowing that some of my brother practitioners at other agencies were also short of anti-periodic remedies, I at once reported my successes to my friend Dr. Irving W. Smith, physician to the Kiowa and Comanche Agency. He reported some time after—"I have tried the new remedy in a number of instances, with both red and white patients, in each instance with complete success as far as reported." "I have only a few ounces of quinia or cinchonidia, and regard the new remedy as a special blessing at this time." "I have added the tinct. to simple syrup, a drachm to the ounce, with enough iodide of potassium to prevent precipitation on the addition of water."

I also reported upon the value of Iodine as a substitute for quinia to the "Cincinnati Lancet and Clinic," and several succeeding numbers contained testimonials from various parts of our country as to its decided value. Since coming to Tennessee I have had fewer cases of a malarious character with which to deal, but in these few, I am perfectly satisfied with its results, as they have been fully equal to those recorded in my government practice.

Several practitioners along the Tennessee River, where miasmatic fevers are more prevalent—have, at my suggestion, used the iodine, and they too have been astonished at the favorable results. In one instance the doctor informed me that he had used quinia and the various alkaloids of Cinchona without avail in an obstinate case of malarial fever in a child, that he had also tried arsenic and other antiperiodic remedies of repute, but without success, that he finally prescribed the iodine, with a perfect cure as the result. Previous to this he said he had so little faith in the remedy, (although it had been proposed to him some time before this) that he had not tried it, and only beeause of want of success in the use of all other remedies had he tried it in this instance, but he now proposed to give it a further trial.

The fact that the cost of iodine is so little in comparison to quinia, especially renders it a boon to the poorer classes, who can ill afford to purchase quinia, and the physician who treats them and furnishes the remedy, finds his bill for drugs not an insignificant item of his expenses, with little to show in return.

The fact that iodine exerts such a pronounced effect upon those very glands which appear to harbor the malarial poison, seems to render its great value in these cases, quite philosophical.

It only seems strange, that in the settling up of our great West, with malarial poison ever opposing the onward march of civilization, and the great scarcity and high price of quinia, that the value of this remedy had not been earlier and more generally appreciated.

It is safe to say that hundreds of persons have died of malarial disorders, especially in the Territories and regions remote from medical supplies, because of the scarcity of quinia, or because it could not be obtained at any price, when perhaps at the same time, iodine in quantity was in stock in the dispensaries.

[Read before the Philadelphia County Medical Society, May 12, 1880.]

### RAPID BREATHING AS A PAIN-OBTUNDER IN MINOR SURGERY, OBSTETRICS, THE GENERAL PRACTICE OF MEDICINE, AND OF DENTISTRY.

BY W. G. A. BONWILL, D. D. S., PHILADELPHIA, PA.

What led to the discovery of this effect of rapid breathing was in an operation upon myself in 1855, while inhaling chloroform. I was conscious of touch and not pain. I applied it to dentistry in obtunding sensitive dentine, and finally, in 1875, applied it, by increasing the inhalation to one hundred a minute, to the extraction of teeth, and, soon after, to minor surgery, etc. I can in this way render patients insensible to acute pain from any operation where the time consumed is not over twenty to thirty seconds. While the special senses are in partial action, the sense of pain is obtunded, and, in many cases, completely annulled, consciousness and general sensibility being preserved. To accomplish this each patient must be instructed how to act and what to expect. Simple as it may seem, there is a proper and consistent plan to enable you to reach full success.

Before the patient begins to inhale he is informed of *the fact* that, while he will be unconscious of pain, he will know *full* or partially well every touch upon the person; that the *inhalations must* be rigorously kept up during the *whole operation* without for an instant stopping; that the more energetically and steadily he breathes, the more perfect will be the effect; and that if he cease breathing during the operation the success will not be so complete.

It is very difficult for a person to respire more than one hundred times to the minute, on account of the exhaustion produced. *For the next minute following the completion of the operation the subject will not breath more than once or twice.* Very few have force enough left to raise hand or foot. The voluntary muscles have nearly all been subjugated and overcome by the undue effort at forced inhalation of one hundred over twenty,—the normal standard. It will be more fully understood, further on in my argument, why I force patients, and am constantly speaking to them to go on.

I further claim that for the past four years, so satisfactory has been the result of this system in the extracting of teeth and deadening extremely sensitive dentine, that there is no longer any necessity for chloroform, ether, or nitrous oxide in the *dental* office for such purposes.

The anæsthetics, when used in major operations, where time is needed for the operation, can be made more effective by a lesser quantity when given in conjunction with "rapid breathing." Drs. Garetson and Hewson, who have thus tried it, tell me it takes one-half to three-fourths less; and the after-effects are far less nauseating and unpleasant.

As an agent in labor where an Anæsthetic is indicated, it is claimed by one who has employed it (Dr. Hewson) in nearly every case for three years, that he has used "rapid breathing" solely and to the exclusion of chloroform and ether.

If in breathing the quantity of carbonic acid gas set free is in exact relation to the amount of oxygen taken into the blood, what effect *must be* manifested where one hundred respirations in one minute are made while the heart is propelling the blood only a very little faster through the lungs, and *more feebly*,—say ninety pulsations at most,—when to be in proportion it should be four hundred to one hundred respirations to sustain life any length of time?

You cannot deny the fact that a definite amount of oxygen can be absorbed, and is absorbed, as fast as it is carried into the lungs, even if there be one hundred respirations to the minute while the pulsations of the heart are only ninety. Nature has *made it* possible to breathe so rapidly to meet an emergency, and we can well see its beautiful application in the normal action of both the heart and the lungs while one is violently running.

You are already aware how small a quantity of carbonic acid in excess in the air will seriously effect life. Even two to three per cent. will in a short time prove fatal. In ordinary respirations of twenty to the minute the average of carbonic acid exhaled is 4.35.

From experiments long ago made by Vierordt (see Carpenter, p. 524) you will see the relative percentage of carbonic acid exhaled from a given number of respirations. When he was breathing six times per minute, 5.5 per cent. of the exhaled air was carbonic acid; twelve times, 4.2 per cent.; twenty-four times, 3.3 per cent.; forty-eight times, 3.0 per cent.; ninety-six times, 2.6 per cent.

Let us deduct the minimum amount—2.6 per cent. of carbonic acid when breathing ninety-six per minute—from the average, at twenty per minute, or the normal standard, which is recorded in Carpenter (p. 524) as 4.35 per minute, and we have retained in the circulation nearly two per cent. of carbonic acid ; that, at the average, would have passed off through the lungs without any obstruction, and life equalized ; but not having been thrown off as fast as it should have been, it must of necessity be left to prey upon the brain and nerve-centres ; and as two to three per cent., we are told, will so poison the blood, life is imperilled, and that speedily.

I think we are now prepared to show clearly the causes which effect the phenomena in "rapid breathing."

The first thing enlisted is the *diversion of the will-force* in the act of forced respiration at a moment when the heart and lungs have been in normal reciprocal action (twenty respirations to eighty pulsations), which act could not be made and carried up to one hundred respirations per minute without such concentrated effort that ordinary pain could make no impression upon the brain while this abstraction is kept up.

Second.—There is a specific effect resulting from enforced respiration of one hundred to the minute, due to the *excess of carbonic acid gas set free from the tissues*, generated by this enforced normal act of throwing into the lungs *five times* the normal amount of oxygen in one minute.

Third.—Hyperæmia is the last in this chain of effects, which is due to the excessive amount of air passing into the lungs, preventing but little more than the normal quantity of blood from passing from the heart into the arterial circulation, but damming it up in the brain.

The Obstetric Treatment of Perineum.—In the above paper Dr. Garrigues, of this city, gives a description of the perineum and external organs of generation, at the same time combating many errors that prevail in reference to the anatomy of the parts. In speaking of lacerations, he says that ergot should not be given until after expulsion of the placenta, as it hastens the delivery before there is sufficient dilatation of the obstetric canal, and may result in laceration. He also advises, as means of lessening the chances of this accident occurring, that the rectum shall be thoroughly evacuated by an enema of warm water. He advocates the support of the perineum, but not until the presenting part has commenced to dilate the vulvar orifice. In the intervals between the pains a kind of enucleation may be performed by passing the index and middle finger of the left hand into the rectum and pressing against the forehead of the child, thus aiding in the expulsion of the fœtus. When the shoulders are passing through the external orifice, he claims that if the posterior one be pushed backward, or the anterior pulled forward by means of the index finger hooked in the axillæ, perineal laceration may, in many instances, be avoided, as he has many times examined the perineum before delivery of the body, and found no laceration, whereas it existed afterward. When the vaginal orifice is too small, he recommends episiotomy, which can be performed by cutting the constrictor vaginæ muscles on each side. All these methods of procedure, according to the circumstances existing in each case, are recommended by Garrigues, to avoid, if possible, a perineal laceration. If a laceration takes place which is slight, he employs serres-fines, one to three being used. They are applied as follows: the lips of the wound are raised up by means of the thumb and index finger of the left hand, and the serrefines applied with the right, commencing with that portion of the laceration near the anus. They are allowed to remain about four days, when they are removed. He speaks very highly of them, because of the ease with which they may be applied, and also on account of the small amount of pain occasioned by their application. In extensive lacerations, sutures must be applied, and a description of different methods of suturing is given.—*Reprint from Am. Jour. of Obstet.*, April, 1880.

The National Board of Health, about a year ago, had at its disposal an appropriation of 550,000 dollars. This money is almost gone and an appropriation of 75,000 dollars has recently been called for by a bill which elicited considerable discussion in the House of Representatives. One amendment was proposed, striking out the 75,000 dollars and substituting 125,000 dollars and another substituting 150,000 dollars. After a stormy debate, in which the board was arraigned for extravagance the bill, as originally reported, passed. During the year in which the board consumed this large amount of money no unusual epidemic was reported. How much would it cost to support this National Board of Health during a year of plentiful yellow fever or cholera? The fact that yellow fever did not flourish to any great extent last year is not much evidence that "the board stamped it out" as it claims. The officious and oracular and demonstrative procedures of the board during the past year have not convinced the nation and the profession of its usefulness. Its treatment in the House of Representatives is evidence of this fact and points strongly to the abolition of the board as at present constituted. This may be said, with no reflection upon individual members of the present board, many of whom we know to be of unquestioned ability and integrity.

Manifestly the sanitary interests of a nation demand sanitary government and the requisite power must necessarily be delegated somewhere. Dr. Henry I. Bowditch, in an article published in the Boston Medical and Surgical Journal, January 8, 1880, proposes a reasonable solution of the problem. He would have this power vested in a cabinet officer, the Secretary of Health, who should hold co-ordinate rank with other members of the cabinet; he should reside at the seat of government; he should have the right in great emergencies to summon sanitarians for purposes of consultation. He should also have an advisory council always accessible, for advice and support, and without whose support he could take no important step, and this council together with the cabinet officer should constitute a National Board of Health. This board should confirm all the doings of the secretary and by a two-thirds vote of the whole board should have the power to veto any proposed action of the secretary, in which emergency an appeal to the President should be made, who, with the secretary, should have absolute power. The English government has adopted a similar plan. Such a plan would have all of the advantage of centralization, and, by giving the health officers absolute power, would enable them to act promptly and efficiently in great emergencies. It should be so organized as to render the national health department analogous to the national war department, which has in times of peace a nominal standing army, but which has the capabilities for almost unlimited expansion in the emergency of war. The war department does not in time of peace need 500,000 men to demonstrate its usefulness. Nor does the health department in time of health require a 500,000 dollar demonstration of its usefulness.

Coca as an Antidote for the Opium Habit.—Dr. E. R. Palmer, Professor of Physiology in the University of Louisville, gives (Louisville *Medical News*, May 29,) his experience with this remedy. Of the habit itself, he writes: "De Quincy and others have founded the pernicious notion among the laity that there is something far more divine in the intoxication produced by opium than in the common intoxication of alcohol. * * * * It has been my lot, like that of most practitioners, to come in contact with opium-eaters, and I will positively affirm that I have yet to see one who even approximated in his nature the 'happy-go-lucky' character of the drunkard. Opium-eating is a curse without any qualifying dispensation—a black cloud in a sunless life. Unlike alcohol, it cannot be said of opium that its constant use improves the vital powers of the enfeebled No debate as to its food properties ever have or ever can be held. It is simply a powerful drug, useful in time of great physical distress, and pernicious beyond the power of pen to portray when once it fastens itself upon the mortal frame as a daily necessity."

After stating that there is no remedy, so far as the literature of medicine of the day goes, which has any claim whatever to be regarded as a curative of the habit, he describes his experience with fluid extract of coca in two cases of cardiac irregularity, with hypochondriasis, which got well under its use.

The first case of the opium habit was that of a man whose system had been shattered by taking three grains of morphine several times a day. Various remedies were tried for a day or two, and by moral suasion he was induced to diminish the dose very materially, but much to his discomfort. The following is added: "About the third day of my attention I bethought myself of the coca and ordered it for him. Imagine my surprise upon meeting him the next day with fine spirits and a record of only one-fourth of a grain of morphine taken since my last call. This was the end of the case. He took the coca for some days, and entirely broke off from opium. His statement was that whenever he felt depressed or bad he took a good, big dose of the medicine, and in a few moments was all right."

The second case we cite in the writer's own words: "Upon the 18th of the present month a gentleman sent for me. I found him in bed, looking like a consumptive. He at once told me that he was an opium-eater, and that he had reached a point where thirty grains of morphine daily were necessary to supply the cravings of his perverted nature. He said that he was now trying to break off, and wanted me to help him. I told him what the coca had done, and, with a few cheerful words, prescribed it for him. The next day I found him still taking morphine, although in small doses, as he had not been able to find the coca. Upon the following day he had had but one dose of morphine in eighteen hours, (one-fourth grain,) and plenty of coca. He was hopeful and cheerful. The next day I failed to seem him, and on calling the following day the servant met me at the door with the statement that he was well, and had gone down the street. This much I can say for the last case, that when I last saw him he looked like another man, so light and cheerful was his face and so free from the evidences of opium."

Dr. Palmer admits that these are very brief and slender grounds upon which to base a claim of discovery ; but thinks they are sufficient to warrant the direction of professional attention to the use of coca in the treatment of the opium habit.

"Erythoxylon coca is a native of the eastern slope of the Andes. It is cultivated in the tropical valleys of Bolivia and Peru. The greatest of care is given to its culture by the natives. An idea of its importance as an agricultural product may be gained from the fact that the duties upon coca in Peru amount yearly to four hundred thousand dollars. The Peruvians are pre-eminently a despondent, an unhappy race, and coca is their balm. To them it is a relic of departed days of glory, and under its benign influence they enjoy in dream and delirium the halcyon days of Manco Capac. * * * * * The ordinary dose for adults of the fluid extract is a table-spoonful.

TONGA.—Under the name of "Tonga," a new remedy for neuralgia has recently attracted considerable attention in the medical profession. Some months ago, Mr. Ryder, a gentleman residing in Fiji, brought home a drug used by the Fijians in cases of this malady (*London Medical Record*, April 15th). It was brought in the form of small broken fragments, consisting of a mixture of woody fibre, bark and leaves, broken into such small pieces as to be almost impossible to identify any portion botanically. This broken vegetable matter was tied up into spherical bundles, each about the size of an orange, covered with a wrapper consisting of the fibrous and breathing base of the leaves of the cocoanut palm (*cocos nucifera*). Notwithstanding the broken state of the drug, Mr. G. M. Holmes, the curator of the museum of the Pharmaceutical Society, has (*Pharmaceutical Journal*), after careful examination, arrived at the conclusion that the principal component part of the contents of the bags is the stem of an arvideous plant, a species of *Rhaphidiphora*, probably *R. vitiensis*. The mode of preparing a draught of tonga for use is extremely simple. The bundle, while still closed, is to be allowed to soak in cold water for ten minutes. The liquid is then to be squeezed out into a tumbler, and a claret-glass of the infusion taken three times a day, about half an hour before each meal. The bundle is to be dried and hung up in a dry place, and can be used several times for a year. Experiments made in its administration by Dr. Sydney Ringer and Dr. Murrell, in eight cases, proved successful; in six, it acted very promptly; one was much improved; the other was not affected. The peculiarity of the action of this drug is its rapid action on the nerves; it does not affect the pupil when topically applied, nor increase nor lessen either perspiration or saliva.—*British Medical Journal.*

Night Medical Service.—A bill for the organization of a night medical service in New York city has been introduced into the Legislature. The bill is divided into eight sections. The first provides for the registry, by the captains of the several police precincts, of the names and addresses of all physicians in good and regular standing within their jurisdiction who shall make application for such registry. But such lists must be submitted to the Registrar of Vital Statistics of the Board of Health, whose duty it shall be to pass upon the regularity of each name, and to transmit a certificate to the effect that the physician named therein is in good and regular standing. Upon receipt of such certificate, the name of the physician is posted upon the precinct bulletins, and he becomes liable to night medical calls. The fee is fixed at $3, to be paid by the Cashier of the Board of Health, and the night physician may call an expert, surgical or medical, in council, when the urgency of the case demands it. The expenditure is limited to $3,000 per year. The bill is accompanied by a petition praying for its passage, which is signed by the leading members of the profession.—*The Medical and Surgical Reporter.*

Disinfection.—Recent experiments made under the direction of the International Cholera Commission have shown that the ordinary methods of disinfection are inefficient, and, in practice, they have often failed to arrest the spread of infectious diseases.

As it is impossible to experiment directly upon the *unknown* low organisms, which are thought to be the means of transporting the various infectious diseases, the effects of chlorine and sulphurous acid were studied upon *known* living organisms; the probabilities being thought to be in favor of the theory that complete disinfection should destroy at least all known forms of life, although it may be true that the tenacity of life of the infective matter of various diseases differs, just as the degree of cold necessary to put a stop to yellow fever is much less than that required to arrest the spread of cholera.

Chlorine and sulphur fumes, in sufficient quantity, were found to be efficient in killing insects, fungi, bacteria and infusoria; the objections to chlorine in houses being that it is more costly; that its use is more difficult, and that it destroys metals, textile fabrics and colors.

The burning of ten grams of sulphur for each cubic meter of air-space, tightly closed, was found *not* to kill bacteria, infusoria, or all insects; twenty grams, however, were proved to be sufficient for that purpose. One volume of water, when saturated at 59° F., absorbs thirty-seven volumes of sulphurous acid—enough to kill all the low organisms found in putrid urine.

The following articles were found uninjured after several hours of exposure to an atmosphere in which twenty grams of sulphur had been burned to every cubic meter of air-space; a clock of steel and brass; rusty and clean nails; gold and silver money; a military epaulet; various colored silk articles; a colored rug; calico; down-pillows; a gilt-framed looking-glass; books; water in an uncorked bottle; flour; meat; salt; bread; apples; cinnamon; vanilla; cigars; wall-paper; oil paintings; varnished articles; gas fixtures; a highly polished razor had a slightly cloudy appearance on its upper side, but that was easily rubbed off. The flour and meat were cooked and eaten, and the cigars were smoked, without any abnormal taste or smell being observed; in the bread not all of the observers noticed a slightly acid taste; the inside portion of the apples was unchanged, the skin was slightly sour; the water, after standing, had an acid reaction, but no decided taste or smell. Litmus paper placed between the leaves of books and under the carpet was turned bright red. Many of the articles exposed had a decided smell of sulphur at first, but that soon disappeared.

The experiments seemed to show that clothing, bedding and other articles may be disinfected without being changed chemically or injured; and it should be added that practically this method has apparently accomplished perfect disinfection, as tested in Berlin.

If we may judge from these results, effective disinfection, by burning sulphur, requires eighteen ounces to each space of one thousand cubic feet. The sulphur should be broken in small pieces, burned over a vessel of water or sand, so as to avoid danger from fire, and if the room is large, it should be put in separate vessels in different places. The room should be tightly closed for six hours, and then aired; it is better that the room should be warm than cold. Of course, efficiently disinfected air is, during the process of disinfection, irrespirable. Most articles may be disinfected in this way, if hung up loosely in the fumigated chamber, although it would be an additional safeguard to expose anything thick, like a bed-mattress, to prolonged heat at a temperature of about 240° F.; and indeed, heat must, with our present knowledge, be considered the best disinfectant. With this end in view, local boards of health are advised to procure furnaces and laundries, as is commonly done in other countries, to be used for the sole purpose of disinfecting articles which have been exposed to the infectious diseases, as recommended in the Ninth Annual Report of the State Board of Health, and described by Dr. A. H. Johnson, in an exhaustive paper on scarlet fever. (pp. 255 *et seq.*), in that report. Of course, a much simpler disinfecting furnace than that described will answer every purpose. For ordinary use, in disinfecting *houses*, the sulphur process is the best.

A solution of chloride of zinc (one part of Burnett's Disinfecting Fluid to two hundred of water), very quickly kill bacteria *which have been placed in it*, and arrests putrefaction. Caustic lime serves equally as well (1 to 100), but leaves a sediment not always easy to remove. Carbolic acid in sufficient strength to be effective (1 to 100) is more expensive and of disagreeable odor.

It is needless to add that "disinfectants" used in sufficient quantities to destroy bad smells do not necessarily kill microscopic living organisms; and it is not supposed that they directly influence the so-called "germs" of the infectious diseases, unless concentrated to the extent which has been mentioned.

Finally, fresh, pure air acts as one of the best "disinfectants" by enormously diluting the infectious matter, and, under certain conditions, including time, must render it inert to all effect, even if not quickly destroying it, as many thinks is the case.—*Circular from Mass. State Board of Health.*

Population Statistics of France and Germany. — Some interest attaches to the population statistics of two large countries like France and Germany, and the returns which have recently been published show results which are not without importance. In France the attention of statisticians has of late been directed to the steady and serious decrease in the population, as evinced by the gradual decrease in the birth-rate. In 1878 the number of births was 937,211. The two causes accounting for the decreases are the fewer number of marriages and, what is far more important, the decline in the number of children resulting from these marriages. In fact, the proportion of children to each marriage is dwindling more and more all over the country, except in Brittany, and in some of the departments in the centre and the south. In the class composed of petty tradesmen, or the well-to-do peasants, there is seldom more than one child per marriage, and M. Baudrillart has stated that in one of the rural communes in Picardy he ascertained the number of children among the best-off of the peasants to be thirty-seven for thirty-five families. To a certain extent the decrease in population is kept in check by the decrease in the mortality, which numbered 839,036 in 1878, or 2.26 per cent., whereas in 1864-68 it was 2.34. But the question nevertheless remains as to what will be the ultimate destiny of France if the decline of population still continues uuchecked. In Germany, on the other hand, the estimated population of the Empire at the end of 1878 was calculated in round numbers at 44,211,000; the number of births during that year was 1,785,080, and the deaths 1,228,707, giving an excess of births over deaths of 556,473, or an annual increase of population amouting to more than 1.25 per cent. For every 1,000 of the population 15.4 were married, 40.4 were born, and 27.8 died, so that the births were more numerous than the deaths by 12.6 per 1,000.

ARTIFICIAL RESPIRATION.—The *Medical Press and Circular*, 1880, informs us that in a recent communication to the French Academy, Professor Fort raises again the question of premature interments. One fact he mentions is, that he was enabled to restore to life a child three years old, by practicing artificial respiration on it four hours, commencing three hours and a half after apparent death. Another case was communicated to him by Dr. Fournol, of Billancourt, who, in July, 1878, re-animated a nearly drowned person after four hours of artificial respiration. This person had been in the water ten minutes, and the doctor arrived one hour after asphyxia. Professor Fort insists also on the utility of artificial respiration in cases of poisoning, in order to eliminate the poisons from the lungs and the glands. The length of time it is desirable to practice artificial respiration in any case of apparent death from asphyxia, Professor Fort has not yet determined, but his general conclusion is that it should be maintained perseveringly for several hours.—*Scientific American.*

THE EFFICIENCY OF THE WATER TRAP.—A cotemporay publishes an important experimental investigation by Dr. Neil Carmichael concerning the trap and water closet system, and their relation to sewage products, gaseous and others. As the result of this investigation, Dr. Carmichael came the conclusion that an efficient water trap excludes soil pipe atmosphere to such an extent that what escapes through the water is so little in amount, and so purified by filtration, as to be perfectly harmless. The water trap, he further concludes, stops entirely the passage of all germs and particles from the air of the soil pipe, including the specific germs or contagia of disease, which, so far as is known, are particulate. He thus traverses the belief so largely entertained that the water of a trap, however perfect in arrangement, will absorb the air of the soil pipe until saturated. and then give it off harmfully on the house side. He would rehabilitate the old faith in the sufficiency of the water to insure safety and he would refer the harm from traps to their imperfect sealing, or to various deteriorations in the structure of the water closet or soil pipe which permit direct communication between the air of a house and the air of the soil pipe. The series of experiments on which Dr. Carmichael has founded these conclusions are exceedingly ingenious, and would certainly appear to justify them, but we doubt whether he has been sufficiently careful in indicating the conditions under which the safety of the water trap can be secured.—*Lancet.*

BLEACHING TEETH.—Dr. W. H. Atkinson, D. S., of this city, gives the following directions for treating discolored teeth: To bleach a tooth discolored by loss of its pulp: Carefully clean it out to the end of the root, going *through* the apex into the *always present latent abscess* at the end. After drying out as well as you can, proceed to fill nicely all the length of the canal in the root and the pulp chamber with oxychloride of zinc.

As soon as it is well hardened excavate out all the discolored dentine that can be spared without weakening the tooth. Then fill the nerve chamber with powdered alum, and wet it with Labaraque's solution of chloride of sodium (such as the laundress uses in washing). This will bleach any tooth that is stained by vegetable color. Now dry well and fill with such shade of oxide of zinc as will restore normal color. When hard cut out the surface and cover with gold. In case iron be the color agent, it may be removed by dissolving a few crystals of oxalic acid in the cavity; afterward proceed as before directed.

Faithfulness in following these instructions, the doctor says, will result in satisfaction to patient and practician, by perseverance.

A Case of Melanosis in Philadelphia.—For some months a Philadelphia physician has had under treatment an infant afflicted with the rare disease, melanosis, in an aggravated form. The child was born with a fair complexion, dark eyes, and brown hair. Soon after birth he began to turn dark of skin, the color deepening from yellow to saffron, and finaly to black. The color was uniform all over the body, except at the joints where it was a little darker, and in the palms of the hands where it was lighter. The once brown hair grew stiff and jet black, and the eyes also grew darker, so that the line between the pupils and the iris could not be distinguished.

In spite of medical treatment the boy became worse, and grew very weak, all the time the color of his skin deepening. At last he became as black as a full blooded negro. Then he was attacked by convulsions, which grew more frequent and violent until they threatened the child's life. It was in one of these that Dr. Reynolds was called in. He succeeded in curing the spasms, an then devoted his attention to the strange disease which afflicted the child. He at once recognized it as melanosis or pigmentation, which is mentioned in the books in a general way, but their is no case given where it had developed all over the body. This was more than sixteen months ago, the child being then thirteen months old.

Since then the boy has greatly improved, by degrees becoming lighter, until now he is of a light chestnut brown color. The case has naturally attracted much attention from physicians.

Domestic Enema.—We are informed by Gailard's Medical Journal that the Zulus have a way of their own of cleansing the gastro-intestinal canal, by producing emesis. They place the patient on his head, and, introducing the small end of a cow's horn into his rectum, pour a quart of sea water through it into the recesses of his body. This is what our Illinois State Board of Health would call a "domestic enema." It has the charm of simplicity and the merit of thoroughness, but it is liable to the objection of leaving a bad taste in the mouth.—*Chicago Medical Gazette.*

Chloride of Calcium in the Treatment of Phthisis.—James Sawyer, M. D., M. R. C. P., says, in a communication to the *British Medical Journal*, June 5, 1880—

Have we a remedy for phthisis? We now know that the term chronic pulmonary phthisis includes a variety of pathological conditions and a variety of textural lesions in the lungs which have long been recognized as distinct, which recent research has done much to unravel, and about which we may still expect to learn more. We know the differing clinical and pathological courses of tubercular phthisis, unresolved lobar pneumonia, chronic catarrhal lobular pneumonia, and pulmonary scirrhosis. All these are included in the generic name phthisis. When I say have we a remedy for phthisis? I mean have we a remedy for this allied group of conditions, due to varying pathological changes, but marked in common by progressive wasting of the body, by progressing asthenia, by progressing diminution of respiratory capacity, and by fever of hectic type. Every case of phthisis requires special study, and can be treated by no rule-of-thumb practice because it is phthisis In one case of anæmia is prominent and calls for iron, perhaps for arsenic; in another, continued but small hæmoptysis calls for ergot or hamamelis; in another, a racking and frequent cough calls for opium, morphia, or codeia; in another, dyspepsia calls for alkalies, or acids, or bitters, or pepsine; in another, nervous unrest calls for bromides; in another, laryngeal troubles call for special local medication; in another, we have to aim at controlling excessive sweating or checking an exhausting diarrhœa. Apart from these and other particulars, I suppose we are all agreed that cod-liver oil, given alone, or variously combined with other agents which tend to promote its assimilation, as with ether, as suggested by Dr. Foster, stands at the head of remedies calculated to promote the general nutrition of the phthisical. Have we any other general remedy? For a long time I trusted to syrup of the iodide of iron. This I gave up for a mixture of hypophosphites and iron—five grains of hypophosphite of lime, ten grains of hypophosphite of soda, and fifteen minims of syrup of the phosphate of iron, for a dose. This is a good combination, and I still use it. But chloride of calcium is my favorite drug. I have used it for some years in hospital and private practice, and I believe with great advantage. Perhaps you will say, Do you give it alone? I do not. I give it with cod-liver oil, or with cod-liver oil emulsion, or with morphia, or with ergot; but my general impression is, *quantum valeat,* that I get better results with chloride of calcium with these combinations than I do with anything else in the same combinations. My attention was called to the value of chloride of calcium in phthisis by a paper in one of our medical journals, wherein it was stated that the drug was much used by the late Dr. Warburton Begbie. Scarcely mentioned, if noticed at all, in books on drugs, chloride of calcium has an old repute for the cure of strumous glandular swellings. In phthisis I give ten grains, dissolved in a drachm of water and mixed with a drachm of glycerine, in a wine-glassfull of milk, twice daily, immediately after meals. I think it tends to check night sweats, to cause increase of weight, and to dry up pulmonary lesions. Of course I do not maintain it does these things in all cases. What I have stated are general conclusions, open, I am aware, to objection, on the ground of their insufficient logical basis, but conclusions which have been and are for me grounds of therapeutic conduct. In prescribing chloride of calcium, we must be careful to write the name of the drug distinctly in full, in order to avoid an error from which one of my patients suffered, namely, the substitution of "chloride of lime."—*Medical and Surgical Reporter.*

Measles not a Trivial Disease. — Since January 1, 1880, says "*The Proceedings,*" there have been 1,864 cases of measles reported to the Brooklyn Health Department; this is probably less than half the number which has actually occurred. During the same time there have been 73 deaths from the same disease, while during the entire year 1879, measles caused but 40 deaths; should the present rate of mortality continue throughout the year, the record will show 240 deaths from measles for the twelve months of 1880. While measles has thus far caused 82 deaths, there have been but 65 deaths from scarlet fever. In New York last year, measles caused 244 deaths.

It is a common impression that measles is a trivial disease which every child must have at some period of its life; that the younger he is the more mild the attack, and therefore the sooner he has it the better.

This is the popular opinion, and some physicians hold the same views. From practical local observation and careful investigation of the subject, together with the experience of Brooklyn physicians obtained from their answers to a series of questions sent them by the Board of Health, we believe, says Dr. J. H. Hammond, Sanitary Superintendent, Brooklyn, that the general impressions referred to are entirely erroneous, and if permitted to go uncontradicted, liable to do great harm and injury, even to the degree of sacrificing life.

Action of the Board of Health:

Measles being so prevalent in Brooklyn; and its mortality so great, the Board of Health has included this disease in the same category with scarlet fever and diphtheria, and requires the following action:

1. Reports to be made to the health office by physicians, of all cases coming under their care.

2. The exclusion of the sick and others residing in the same house, from the schools of the city, both public and private, until a permit for their return is obtained from the Board of Health.

3. These permits to be given when the patient is no longer in condition to spread the disease, and when the rooms, clothing, and other infected materials have been properly fumigated.

4. The fumigation prescribed by the Board of Health is by the burning, for five hours, of sulphur, one pound to each thousand cubic feet of space to be fumigated, the apartment being tightly closed.

5. Certificates of physicians that these requirements have been fulfilled will be sufficient evidence, and on their presentation to a sanitary inspector or at the office of the Board of Health, the school permit will be at once issued.

EXTRAORDINARY FECUNDITY.—According to a paragraph in *La France Medicale*, a woman, whose name and address are given, was several months pregnant when she was seized with colicky pains. Attributing them to ordinary causes, she went into her vineyard, and was profoundly astonished to discover presently that she had been confined. Dr. Watering, of Maregnac, was called to her, and found that she had given birth to eight children, perfectly formed. They were inclosed in a sac, and had apparently perished from mutual pressure during their growth. The mother did well.

